# Ecological dynamics and complex interactions of *Agrobacterium* megaplasmids

**DOI:** 10.3389/fpls.2014.00635

**Published:** 2014-11-14

**Authors:** Thomas G. Platt, Elise R. Morton, Ian S. Barton, James D. Bever, Clay Fuqua

**Affiliations:** Department of Biology, Indiana UniversityBloomington, IN, USA

**Keywords:** plasmids, ecology, replicon, genome, bacterial, pathogenesis, virulence

## Abstract

As with many pathogenic bacteria, agrobacterial plant pathogens carry most of their virulence functions on a horizontally transmissible genetic element. The tumor-inducing (Ti) plasmid encodes the majority of virulence functions for the crown gall agent *Agrobacterium tumefaciens*. This includes the *vir* genes which drive genetic transformation of host cells and the catabolic genes needed to utilize the opines produced by infected plants. The Ti plasmid also encodes, an opine-dependent quorum sensing system that tightly regulates Ti plasmid copy number and its conjugal transfer to other agrobacteria. Many natural agrobacteria are avirulent, lacking the Ti plasmid. The burden of harboring the Ti plasmid depends on the environmental context. Away from diseased hosts, plasmid costs are low but the benefit of the plasmid is also absent. Consequently, plasmidless genotypes are favored. On infected plants the costs of the Ti plasmid can be very high, but balanced by the opine benefits, locally favoring plasmid bearing cells. Cheating derivatives which do not incur virulence costs but can benefit from opines are favored on infected plants and in most other environments, and these are frequently isolated from nature. Many agrobacteria also harbor an At plasmid which can stably coexist with a Ti plasmid. At plasmid genes are less well characterized but in general facilitate metabolic activities in the rhizosphere and bulk soil, such as the ability to breakdown plant exudates. Examination of *A. tumefaciens* C58, revealed that harboring its At plasmid is much more costly than harboring it’s Ti plasmid, but conversely the At plasmid is extremely difficult to cure. The interactions between these co-resident plasmids are complex, and depend on environmental context. However, the presence of a Ti plasmid appears to mitigate At plasmid costs, consistent with the high frequency with which they are found together.

## INTRODUCTION TO AGROBACTERIAL MEGAPLASMIDS

Plasmids play a key role in the ecology and evolution of bacterial populations as they frequently carry genes conferring traits such as antibiotic resistance, pathogenesis, and the ability to breakdown nutrients (Turner et al., 2002; Slater et al., 2008; Rankin et al., 2011). These independently replicating genetic elements are primarily distinguished from chromosomes by the defining characteristic of carrying only non-essential genes. In addition they tend to be smaller than bacterial chromosomes and often encode conjugative systems that allow for their horizontal transmission to other bacterial cells (Thomas and Nielsen, 2005; Harrison et al., 2010). Because they often confer phenotypes that are beneficial in particular environments, plasmids and their horizontal transfer have an important role in structuring bacterial communities and in shaping the evolution of bacterial populations (Slater et al., 2008).

Many members of the *Rhizobiaceae* have multipartite genomes that include several ecologically important plasmids (Jumas-Bilak et al., 1998). The genome of *Rhizobium etli* CFN42 provides a particularly dramatic example of this, being composed of a primary chromosome, a secondary chromosome or chromid, and five plasmids (Harrison et al., 2010; Landeta et al., 2011). Many members of the *Rhizobiaceae* family live in intimate association with plant hosts. Some, such as many rhizobia, are nitrogen fixing plant mutualists, while others, like many agrobacteria, are plant pathogens. The taxonomic status of the genus *Agrobacterium* has been debated with proposals that it be considered a species of *Rhizobium* (Young et al., 2001, 2003; Farrand et al., 2003). However, for continuity and clarity in this review we follow the convention of distinguishing between agrobacterial and rhizobial strains.

The rhizobial nitrogen fixation and agrobacterial pathogenesis functions that characterize their association with plants are largely conferred by the plasmids they carry. The conjugal Ti (tumor-inducing) and Ri (root-inducing) plasmids found in many *Agrobacterium* species carry the majority of genes underlying crown gall and hairy root disease, respectively (Escobar and Dandekar, 2003; Suzuki et al., 2009). Ti plasmids are harbored by both generalist pathogens including many *Agrobacterium tumefaciens* strains, and narrow-host range pathogens such as *A. vitis* strains that cause crown gall of grape. Ri plasmids are typically found in pathogenic *A. rhizogenes* strains that cause hairy root disease. Unless indicated otherwise, this review will focus on *A. tumefaciens*, although many of the general features of plasmid biology and plant infection are similar in *A. rhizogenes* and *A. vitis*.

Infection of a plant host involves its genetic transformation in which a large segment or segments (approximately 40 kb) of Ti plasmid-borne genes (the transferred or T-DNAs) are replicated from the plasmid via a conjugation-like mechanism, delivered into the plant cell via a type IV secretion system, and integrated into the host plant’s genome (Escobar and Dandekar, 2003; Brencic and Winans, 2005). Ti plasmid virulence genes are only expressed when pathogenic *A. tumefaciens* cells encounter a specific set of environmental conditions (plant-produced phenolics, sugars, low pH, and limiting phosphate) most indicative of wounded plant tissue (Winans, 1990). Following transformation, the plant host cell machinery directs the expression of T-DNA genes, leading to T-DNA controlled synthesis of the plant hormones auxin and cytokinin, resulting in accelerated division of transformed plant cells (Drummond et al., 1977; Garfinkel et al., 1981). This gives rise to the most conspicuous symptom of crown gall disease—tumor development. Less conspicuously, but arguably of primary importance for the pathogen, the plant’s expression of T-DNA genes also results in the synthesis and release of a suite of unique metabolites that are broadly termed opines (Brencic and Winans, 2005). Opine catabolic genes carried on the Ti plasmid allow the pathogen to catabolize the plant produced opines, providing a key benefit of pathogenesis to the infecting bacteria (Guyon et al., 1993; Savka and Farrand, 1997; Platt et al., 2012b). Hairy root disease caused by Ri plasmid bearing *A. rhizogenes* also involves T-DNA transfer that causes plants to produce opines, however rather than cause tumor development this disease stimulates the growth of adventitious roots. Many Ri and Ti plasmid T-DNA genes show homology, such as the Ti encoded auxin biosynthesis genes, *iaaM* and *iaaH*, and the corresponding Ri encoded *aux1* and *aux2* genes. However, several Ri plasmid T-DNAs genes show limited or no homology to genes found on Ti plasmid T-DNAs. These genes, such as *rolA*, *rolB*, and *rolC*, function in stimulating meristem formation, a key feature distinguishing hairy root and crown gall diseases (reviewed by Britton et al., 2008).

In addition to the well-studied agrobacterial virulence plasmids, agrobacteria can also harbor several less well characterized plasmids (Currier and Nester, 1976; Merlo and Nester, 1977; Albiach and Lopez, 1992). For example, some avirulent agrobacteria in nature harbor opine catabolic plasmids, which confer the ability to freeload on the benefits of pathogenesis initiated by virulent agrobacteria by catabolizing public goods in the form of opines (Merlo and Nester, 1977; Wabiko et al., 1990; Dessaux et al., 1998; Wetzel et al., 2014). The biocontrol agent *A. radiobacter* K84 is the best characterized avirulent strain harboring such a plasmid. Interestingly, K84 also produces several antimicrobials that allow it to interfere with the growth of virulent agrobacteria (Donner et al., 1993; Penyalver et al., 2001; Kim et al., 2006). For these reasons, K84 has served as a powerful commercial biocontrol agent of crown gall disease for several decades. K84’s ability to catabolize opines and produce anti-agrobacterial molecules largely depends on plasmid-borne genes. Wild-type K84 harbors three plasmids. One of these, pAtK84b, confers the ability to catabolize nopaline and agrocinopine produced by crown gall infected plants and shares regions of homology with several Ti plasmids (Oger and Farrand, 2002). A second plasmid, pAgK84, carries genes underlying production and immunity to agrocin 84 (Kim et al., 2006), while a third plasmid, pAtK84a, encodes production of and resistance to agrocin 434 (Donner et al., 1993; McClure et al., 1998). Agrocin 84 specifically inhibits the growth of agrocinopine catabolic agrobacteria such as strains harboring a nopaline-type Ti plasmid (Reader et al., 2005; Kim et al., 2006). In contrast, agrocin 434 primarily inhibits the growth of biovar 2 agrobacteria, the same biovar as K84 itself (Donner et al., 1993).

Tartrate is a common nutrient present on grapevines and many *A. vitis* strains harbor a tartrate utilization plasmid, called pTr or pTar that allows them to access these nutrients (Burr and Otten, 1999). These plasmids likely provide a competitive advantage to *A. vitis* strains in colonizing their grapevine hosts (Salomone et al., 1998). Interestingly these conjugative tartrate utilization plasmids are diverse, though they harbor similar TAR regions required for the degradation of tartrate.

Several pathogenic and avirulent strains of *A. tumefaciens* carry another type of agrobacterial megaplasmid. Like the Ti plasmids, these At plasmids vary widely in their gene structure and composition, though they also share regions of homology. Non-essential for pathogenesis, the At plasmids have received considerably less attention than Ti plasmids. For this reason, they were traditionally referred to as cryptic plasmids as they were previously uncharacterized relative to the Ti plasmids. Although dispensable for virulence, the full sequence of the best characterized At plasmid, pAtC58, reveals the presence of genes involved in a range of functions including, but not limited to, chemotaxis, iron uptake, DNA damage repair, heat shock, and catabolism (Goodner et al., 2001; Wood et al., 2001; Slater et al., 2009). One set of At plasmid genes that has received particular attention are the *blcABC* genes, previously named *attKLM,* because of their initially proposed, but later refuted role in attachment (Matthysse et al., 2008). We now know, however, that attachment is largely mediated by chromosomally encoded genes (Tomlinson and Fuqua, 2009; Li et al., 2012; Xu et al., 2013), and that the products of the *blcABC* operon confer the ability to catabolize γ-butyrolactone (GBL), plant-released compounds often present at high levels in the soil and rhizosphere (Carlier et al., 2004; Khan and Farrand, 2009). In addition to GBL utilization, At plasmids confer catabolic functions that are likely to contribute to the success of *A. tumefaciens* cells inhabiting the rhizosphere. These catabolic systems include those for degradation of deoxyfructosyl-glutamine (DFG), mannopine (MOP), succinyl semialdehyde (SSA), γ-hydroxybutyrate (GHB), and γ-aminobutyric acid (GABA), and are discussed in greater detail in Sections “The Costs and Benefits Associated with the Ti and At Plasmids” and “Ecological Context of Ti and At Plasmids” of this review.

In addition to catabolic functions, there are several studies demonstrating that At plasmids can affect virulence (Nair et al., 2003; Morton et al., 2013). One such study shows that in some strains the presence of an At plasmid corresponds to an increase in the size of tumors, suggesting a positive impact on virulence (Nair et al., 2003). However, variants of pAtC58 from *A. tumefaciens* C58 have been shown to exhibit differential effects on the expression of pTiC58-encoded virulence (*vir*) genes (Morton et al., 2013). For example, whereas a truncated form of pAtC58 (ΔAtu5207–Atu5408) has a repressive effect on *vir* gene expression, the full-length form of the plasmid had no such effect. The basis for the differences between these studies remains unclear.

In this review, we will focus on the genomic, ecological, and evolutionary significance of the two best-studied agrobacterial plasmids, the Ti and the At plasmids. We will first describe what is known about the function and regulation of replication, partitioning, and conjugation of these plasmids. Then we will discuss their diversity, ecology, and the genomic context of their evolution. Throughout, we will focus on the influence of the biotic and abiotic environmental conditions on the regulation of plasmid encoded genes and how this relates to the ecological costs and benefits associated with these plasmids. Finally, we will discuss how these genetic and ecological factors couple together to influence the evolutionary dynamics of these plasmids.

## Ti PLASMIDS AND THE OPINE CONCEPT

Many agrobacterial plasmids are defined by their role in pathogenesis, as is the case for the Ti and Ri plasmids. The Ti and Ri plasmids are highly diverse and are characterized by the type of low molecular weight resources, the opines, that infectious agrobacteria cause host plants to produce. Opines are found within and around the tumors or root hairs of plant tissue that has been transformed by the T-DNA of pathogenic agrobacteria, but are not typically found in soil environments. The range of opines that are produced by the infected plant is determined by the T-DNA genes carried on the virulence plasmid, and these genes vary among strains of agrobacteria (Moore et al., 1997). The driving selective benefit for agrobacterial pathogenesis was for many years proposed to be access to the relatively exclusive opine nutrients (this was known as the “opine concept”), and subsequent experiments demonstrated that this is in fact correct (Dessaux et al., 1998).

As a family, opines are incredibly diverse with over 30 species having been characterized (Dessaux et al., 1998). Chemically, they can be separated into two structural classes: agrocinopines and secondary amine derivatives. Agrocinopines are sugar-phosphodiesters and thus represent sources of carbon and phosphorus (Oger and Farrand, 2002). The amine-derived opines are formed by the condensation of an amino acid with either a sugar or an alpha-keto acid and serve as sources of carbon and nitrogen. Amine-derived opines, such as nopaline and octopine are formed by the reductive condensation of arginine with α-ketoglutarate and pyruvate, respectively. Mannityl opines are derived from deoxyfructosyl-glutamine (DFG), the conjugation product of glutamine and a sugar (Baek et al., 2003).

Opines are usually degraded by catabolic functions that are also Ti plasmid encoded and the expression of which is inducible by the corresponding type of opine. Depending on the Ti plasmid, an *A. tumefaciens* strain can transform plants with one or more of a multiple array of opine biosynthetic genes (Dessaux et al., 1998). Corresponding opine uptake and catabolism genes are located on the non-transferred portion of the infecting Ti plasmid (Guyon et al., 1993). The transferred opine biosynthesis genes include those that function to conjugate plant-synthesized products to amino acids, creating additional substrates that can be utilized by infecting cells (Kemp et al., 1979; Hack and Kemp, 1980). Some strains of *Agrobacterium* exhibit chemotaxis toward specific opines. Chemotaxis is the directed movement of bacterial cells determined by chemical gradients (e.g., nutrients) in the environment. For *A. tumefaciens*, opine-specific chemotaxis depends upon the Ti plasmid and as such, correlates with the specific opine biosynthetic and catabolism genes encoded by the plasmid (Kim and Farrand, 1998).

Subsets of the opines, called conjugal opines, mediate horizontal transfer of the Ti plasmid from one bacterial cell to another. This occurs via activation of expression of the gene encoding the LuxR-type transcription factor TraR (described in more detail in the next section). Octopine is the conjugal opine for octopine-type plasmids, and agrocinopine A and B are the conjugal opines for nopaline-type plasmids (Farrand, 1998a). In at least one plasmid, mannityl opines activate *traR* expression and can function as conjugal opines (Wetzel et al., 2014). This process of plasmid transfer depends on the presence of specific opines produced by transformed plant cells as they enable a response to the self-produced diffusible acyl-homoserine lactone (AHL) quorum sensing signal (Zhang et al., 1993; Fuqua and Winans, 1994). Although conjugal transfer of Ti plasmids is completely dependent on the presence of the conjugal opines, the precise regulatory mechanisms vary for each Ti plasmid (Farrand, 1998b). For example, in nopaline-type plasmids, when opines are absent, the conjugation genes (*tra* and *trb*) are actively repressed by the agrocinopine-responsive transcriptional regulator, AccR (Kim et al., 2008). When opines are present, however, and cells are at a population density at which AHL molecules reach inducing levels, transcription of conjugation genes is derepressed. Similar stimulation of conjugation gene expression is mediated through the octopine-responsive transcriptional activator OccR for octopine-type plasmids. In either case, the control of Ti conjugation genes is indirect, and the opines function by elevating the expression of the *traR* gene (Piper et al., 1993; Fuqua and Winans, 1994). TraR directs the process of quorum sensing and is activated and stabilized by forming a complex with accumulating AHL molecules, resulting in the up-regulation of the *tra* and *trb* genes, as well as increased copy number of the Ti plasmid (White and Winans, 2007).

## STABILITY, REPLICATION, AND PARTITIONING OF *repABC* REPLICONS

Low-copy number plasmids require efficient replication and partitioning in order to ensure their efficient transmission during the reproduction of bacterial cells. Many of the large, low-copy number plasmids and secondary chromosomes found in the genomes of agrobacteria and other alphaproteobacteria belong to the *repABC* family of replicons. The transcriptional and post-transcriptional regulation of the *repABC* operon gene products plays a central role in the replication and partitioning of this family of replicons. In this paper, we will briefly describe the regulation of *repABC* replicon replication and partitioning. We will focus on the well characterized regulation employed by the Ti plasmid and describe how this relates to the quorum sensing dependent regulation of Ti plasmid conjugation. Several recent reviews cover these topics in detail (Mazur and Koper, 2012; Pinto et al., 2012).

The primary replication factor RepC and a nearby replication origin to which it binds are required for replication of *repABC* replicons, whereas RepA and RepB proteins coordinate replicon partitioning during multiplication of the bacterial cell. Unlike many plasmids, the partitioning (*repA* and *repB*) and replication (*repC*) genes of *repABC* replicons are typically expressed as one transcriptional unit controlled by a promoter region upstream of *repA*. RepC does not belong to a known larger protein family and the *repC* gene has only been observed in alphaproteobacteria (Pinto et al., 2012). In contrast, the RepA and RepB proteins belong to the family of ParA and ParB proteins, respectively, which includes proteins mediating the partitioning of chromosomal, prophage, and plasmid replicons. At least one agrobacterial tartrate utilization plasmid, pTar, employs a partitioning system belonging to this larger ParA-ParB family (Kalnin et al., 2000).

The multipartite genome of *A. tumefaciens* C58 is composed of a circular *oriC*-type chromosome and three *repABC* family replicons: a linear chromosome, pTiC58, and pAtC58 (Li and Farrand, 2000; Goodner et al., 2001). The replication origins of all four C58 replicons tend to generally localize to the polar region of the cell, although each site is distinct from the other replicons, suggesting that they may be targeted to distinct addresses (Kahng and Shapiro, 2003). This may contribute to the compatibility of these *repABC* replicons or reflect the mechanism(s) that allows for their stable coexistence within a bacterial cell. The location of centromere-like *par* sites composed of one or more palindromic sequences varies among *repABC* replicons. These sites play a key role in plasmid stability, partitioning, and incompatibility as they are thought to be the site where the partitioning machinery binds. In the cases of pTiC58 and pTiR10 these *par* sites are located between *repA* and *repB* within the *repABC* operon, while for other *repABC* replicons *par* sites can be found close to the *repC* stop codon or upstream of *repA* (Cervantes-Rivera et al., 2011; Pinto et al., 2012). Though not found in all *repABC* replicons, pTiC58 and pTiR10 both encode a *repD* gene located between *repA* and *repB* that overlaps the *par* sites of these plasmids (Chai and Winans, 2005a).

The transcriptional and post-transcriptional control of the replication, partitioning, and conjugation of the octopine-type Ti plasmid pTiR10 is particularly well characterized. The *repABC* operon of pTiR10 is influenced by four promoters upstream of *repA* (Pappas and Winans, 2003a,b). RepA of pTiR10 binds to an operator located downstream of the most proximal of these promoters (P4) thereby antagonizing transcription of the operon. The binding of pTiR10 RepA to this operator is thought to be enhanced by formation of a complex with RepB (Pappas and Winans, 2003b). Similarly, pTiR10 RepB binds to the *par* sites within *repD* and this binding is enhanced by the presence of RepA. Taken together, these results suggest that a RepA–RepB complex may bind both the P4 promoter upstream of *repA* and the *par* site downstream of *repA* forming a large double-stranded DNA loop structure involved in the repression of the *repABC* operon (Chai and Winans, 2005a).

The role of RepC in promoting the replication of the Ti plasmid is thought to depend on its ability to bind the replication origin (Pinto et al., 2011). As with many *repABC* replicons, pTiR10 also contains a gene between *repB* and *repC* which encodes an antisense RNA that down-regulates the expression of *repC* (Chai and Winans, 2005b). In the case of pTiR10 this gene is called *repE* and is thought to duplex with the *repABC* transcript in a way that promotes translational termination near the *repC* start codon. This post-transcriptional control, along with transcriptional autorepression of the *repABC* operon mediated by RepA–RepB complexes, likely helps maintain the low copy number state of pTiR10 under many environmental conditions.

The presence of two types of plant-produced molecules, phenolic compounds and opines, stimulates the transcriptional activity of the pTiR10 *repABC* operon leading to higher plasmid copy number when either of these plant cues are present. The opine effect is indirect, through the TraR quorum sensing mechanism, whereas the phenolic induction is mediated by the VirA-VirG two component *vir* gene regulation system. The sensor kinase VirA phosphorylates VirG in response to the presence of plant-produced phenolic compounds. Phospho-VirG binds to an upstream *vir*-box, stimulating transcription from promoter P4 of the *repABC* operon leading to the elevation of plasmid copy number to approximately four copies per cell (Cho and Winans, 2005). The VirA–VirG two component system similarly promotes *vir* gene transcription directing interkingdom gene transfer (Winans, 1991).

As described above, opine-dependent gene regulation can function through repression or activation mechanisms. For example, for octopine-type Ti plasmids such as pTiR10 the transcription of opine transport and catabolic genes is stimulated by the binding of complexes between the LysR-type transcriptional regulator OccR and octopine, one of the opines that this class of Ti plasmid engineers plants to produce (Wang et al., 1992; Fuqua and Winans, 1996). Nopaline-type Ti plasmids, typified by pTiC58, also increase expression of opine transport and catabolic genes in response to the presence of opines. Nopaline uptake and catabolism are activated by NocR, a LysR-type regulator that functions similarly to OccR (von Lintig et al., 1994). However, the best studied example of opine-responsive gene regulation for pTiC58 is derepression of agrocinopine uptake and catabolic genes by AccR, a LacI-type repressor (von Bodman et al., 1992; Kim and Farrand, 1997).

In addition to stimulating opine catabolic functions, the presence of opines in the plant tumor environment also indirectly controls Ti plasmid copy number and conjugation by inducing the expression of the quorum sensing transcriptional activator TraR, encoded on the Ti plasmid (Pappas, 2008). The inducing ligand for TraR is *N*-3-oxo-octanoyl-L-homoserine lactone (3-oxo-C8-HSL), an AHL signal molecule that is synthesized by the activity of *traI*, an AHL synthase also encoded by the Ti plasmid. TraR–AHL complexes stimulate transcription of the pTiR10 *repABC* operon from all four upstream promoters, resulting in a seven- to eightfold increase in plasmid copy number (Li and Farrand, 2000; Pappas and Winans, 2003a). Under the same conditions, TraR–AHL binds another nearby *tra* box stimulating transcription of the divergently oriented *traI*-*trb* operon that controls expression of both *traI* and genes involved in mating pair formation (Mpf) functions required for conjugation of the Ti plasmid. The *trb* operon includes two genes, *trbJ* and *trbK*, which encode entry exclusion proteins that inhibit conjugal delivery of a Ti plasmid into the cell (Cho et al., 2009). In addition, another pair of divergent operons encoding the DNA transfer and replication functions (Dtr) elsewhere on the Ti plasmid are activated by TraR–AHL binding to an intergenic *tra* box. The Dtr functions include the conjugal nickase (TraA) and the coupling factor TraG.

While the replication, partitioning, and conjugation of the Ti plasmid are well studied, much less is known about other agrobacterial megaplasmids. In some cases, there are parallels between the conjugation of the Ti plasmid and these other plasmids. For example, AccR-dependent opine responsive regulation influences the expression of both pTiC58 and pAtC58 conjugal machinery, revealing a mechanism that promotes co-transfer of these plasmids (Lang et al., 2013). Further, the opine catabolic plasmid of K84, pAtK84b, employs opine-dependent quorum sensing to regulate its conjugation. However in contrast to most Ti plasmids, two distinct types of opines can independently induce the conjugation of this pAtK84b, with each inducing the expression of separate and functional *traR* paralogs encoded by the plasmid (Oger and Farrand, 2002). Though the frequency with which this occurs is unknown, one study has documented the transfer of pAtK84b into pathogenic agrobacteria under natural plant-tumor conditions (Vicedo et al., 1996). In this case, the K84 opine catabolic plasmid likely displaced the resident, incompatible Ti plasmid and the concurrent delivery of pAgK84 essentially converted a pathogenic agrobacterial strain into an avirulent, agrocin 84-producing competitor of the pathogen (Vicedo et al., 1996).

Within the same crown gall tumor, these researchers also observed transfer of a Ti plasmid into the K84 background, with likely subsequent recombination between the Ti plasmid and the resident pAtK84b (Vicedo et al., 1996). In this case, conjugation essentially converted the avirulent biocontrol agent into a pathogenic strain that is resistant to biocontrol by K84 and related strains, an outcome that may undermine the long-term efficacy of biocontrol by K84 (Lopez-Lopez et al., 1999). Other studies have reported the origin of pathogenic, agrocin 84 producing strains via the transfer of pAgK84 into pathogenic agrobacteria, demonstrating another threat to the utility of K84 as a biocontrol agent (Vicedo et al., 1993; Stockwell et al., 1996; Raio et al., 2009). Because of this issue, a genetically engineered derivative of K84 in which the conjugal functions of pAgK84 have been disrupted is also available for biocontrol (Jones and Kerr, 1989; Vicedo et al., 1993; Penyalver et al., 2000). These examples illustrate the recombinational modularity of the agrobacterial megaplasmids, and hint at the evolutionary histories that have led to their complex architecture and regulation.

Many plasmids encode toxin-antidote loci, which can promote their stability in growing bacterial populations and mediate within-bacterium competition among plasmids co-infecting the same bacterial cell (Gerdes et al., 2005; Van Melderen and De Bast, 2009; Cooper et al., 2010). Toxin-antidote systems are widespread among bacteria and highly diverse (Van Melderen and De Bast, 2009). Generally toxin-antidote systems involve two linked loci, one encoding a toxic factor and the other an antidote factor that prevents the toxic effects of the first factor from manifesting. Because of this, these systems can lead to the inhibition of daughter cells that do not inherit the toxin-antidote locus, thereby preventing the spread of cells lacking this locus—such as plasmid free cells—through the population (Gerdes et al., 2005). Plasmid-encoded toxin-antidote loci can similarly mediate competition between co-infecting incompatible plasmids by making it difficult to displace the resident plasmid (Cooper and Heinemann, 2000; Cooper et al., 2010). The stability of two nopaline-type Ti plasmids, pTiC58 and pTi-SAKURA, is greatly enhanced by the presence of the toxin-antidote systems they encode (Yamamoto et al., 2007, 2009). The At plasmid of C58 is highly stable despite the high selective pressure favoring lineages that lose the plasmid. This stability may reflect the effects of one or more of the putative toxin-antidote systems that this plasmid encodes (Morton et al., 2013).

Other agrobacterial plasmids encode secreted toxins that are able to mediate interference competition in addition to potentially contributing to plasmid stability. For example, the pAtK84a plasmid confers not only the ability to produce agrocin 434, which antagonizes other biovar II agrobacteria, but also resistance to the toxin (Donner et al., 1993). The same is true for the agrocin 84 plasmid, pAgK84, in that the plasmid confers the ability to produce a toxin as well as resistance to the toxin (Slota and Farrand, 1982; Ryder et al., 1987). Agrocin 84 interferes with cellular leucyl-tRNA synthetase thereby disrupting the translation of agrocin 84 sensitive strains. Importantly, pAgK84 encodes a variant leucyl-tRNA synthetase which imparts resistance to the toxic effects of agrocin 84 (Reader et al., 2005; Kim et al., 2006).

## PLASMID REARRANGEMENTS AND DIVERSITY

The *repABC* family of agrobacterial plasmids is incredibly diverse (Cevallos et al., 2008). These plasmids have interspersed regions of high sequence similarity, suggesting that multiple recombination events have shaped their structure (Farrand, 1998b). These conserved blocks of sequence can be found between plasmids of different strains of the same bacterial species, but also across species, genera, and families (Farrand, 1998b; Galardini et al., 2011).

Although opine biosynthesis genes (located on the T-DNA) and opine uptake and catabolism genes are often linked together on the same plasmid, this is not always the case (Merlo and Nester, 1977). There are several examples of plasmids that lack the virulence functions all together, but still retain the genes for opine transport and catabolism. pAtK84b and pAtK112 are two examples of these plasmids, conferring the ability to catabolize nopaline and agrocinopines. Expression of a gene on the At plasmid of *A. tumefaciens* R10 is required for complete catabolism of octopine (Cho et al., 1996). Another At plasmid, pArA4, found in *A. rhizogenes* is a catabolic plasmid which provides the ability for its host bacteria to utilize MOP, mannopinic acid, and agropinic acid as sole sources of carbon (Guyon et al., 1993).

Additionally, although most Ti plasmids in *Agrobacterium* species are considered to be virulence elements, they exhibit blocks of high sequence similarity to the symbiosis plasmids of other rhizobia (e.g., pRetCFN4d of *Rhizobium etli* and pSymA of *Sinorhizobium meliloti*). Interestingly, the conjugal pili proteins of many of these symbiotic plasmids exhibit homology to the VirB proteins encoded on the Ti plasmid (Chen et al., 2002; Ding and Hynes, 2009). However, despite the similarity between the symbiotic plasmid Type IV secretion (T4S) systems and those encoded on the Ti plasmid, the horizontal transmission of these symbiotic plasmids is regulated by the *rctAB* repression system, distinct from the quorum sensing control of Ti plasmid conjugal transfer genes and the plant-signal dependent expression of the *vir* T4S (Brencic and Winans, 2005; Perez-Mendoza et al., 2005).

Large-scale deletion events have been characterized in the pAtC58 plasmid of *A. tumefaciens* C58 (Morton et al., 2013). A series of repeat sequences (9–13 bp) was found to be distributed within the At plasmid, immediately flanking sites of large deletions (up to 0.19 Mb). These deletions were discovered in several laboratory stocks of *A. tumefaciens* C58, indicating that they occur during normal passaging. The repeated elements are not within known transposable elements and the longer repeats (11–13 bp) are more abundant on the At plasmid than on other *A. tumefaciens* C58 replicons. Strains carrying At plasmids that have incurred these deletions have increased *vir* expression and possess a higher relative fitness in lab culture compared to strains with the full length plasmids, likely due to high carriage costs associated with these deleted segments (Morton et al., 2013). A large majority of genes within the deletion intervals are represented by ABC transporters, so it could be that passage in laboratory culture favors loss of costly genes that would otherwise confer benefits specific to the natural rhizosphere environment. The variety of rearrangements in the At plasmid of *A. tumefaciens* C58 indicates that the replicon is highly adaptable and dynamic. Additionally, the repeats found flanking deletion sites are not present in the closely related *S. meliloti* (Morton et al., 2013), suggesting that they could be specific to *A. tumefaciens* and provide a mechanism for genomic plasticity.

The At plasmid of *A. tumefaciens* ANT4 has also been shown to cointegrate with the Ti plasmid (Vaudequin-Dransart et al., 1998). During matings between *A. tumefaciens* ANT4 and a plasmidless recipient, C58.00RS, some transconjugants possessed a single large replicon containing genes from both the At and Ti plasmids. It was proposed that integration of these replicons can cause gene disruption and potentially inactivate virulence functions through recombination into required regions (Vaudequin-Dransart et al., 1998). Cointegration between these replicons could have effects on the catabolic potential of *A. tumefaciens* strains, as there are numerous potential cointegration sites between the plasmids in separate isolates, in some cases disrupting opine-utilization functions. Similar cointegration events have been described previously in other members of *Rhizobiaceae* (Flores et al., 2000; Mavingui et al., 2002; Guo et al., 2003). In these cases, cointegration was shown to occur between all chromosomal replicons, but did not have strong effects on fitness or symbiotic-proficiency. The significance of cointegration between the At and Ti plasmids with respect to rhizosphere metabolism and pathogenesis remains unclear.

## THE COSTS AND BENEFITS ASSOCIATED WITH THE Ti AND At PLASMIDS

Plasmids can impose significant fitness costs on the bacterial cells that harbor them (Slater et al., 2008; Baltrus, 2013), which along with the benefits that plasmids confer play a central role in determining their ecological and evolutionary dynamics (Slater et al., 2008). The net balance of these costs and benefits determine whether a genotype with the plasmid has an advantage or disadvantage relative to a competitor genotype lacking the plasmid. Plasmid costs can result from a variety of causes, including the energetic burden of plasmid maintenance or conjugation as well as the costs associated with expressing other functions encoded by the plasmid (reviewed in Baltrus, 2013). These different forms of plasmid costs may vary in their magnitude and degree of context-dependence. The fitness cost of harboring two Ti plasmids, the octopine-type pTi15955 and the nopaline-type pTiC58, has been demonstrated to be low or even undetectable in their respective host backgrounds under laboratory conditions (Platt et al., 2012a; Morton et al., 2014). These low carriage costs likely reflect the tight gene regulation that controls expression of most genes on the Ti plasmid. Natural selection acting on plasmid genes likely favors this tight regulation, as high carriage costs antagonize the fitness of plasmid and chromosomal genes alike. Carriage of pTi15955 conferred a small competitive disadvantage against plasmidless derivatives; however, this was only measurable when the bacteria were limited for either carbon or nitrogen (Platt et al., 2012a). In contrast, when the bacteria competed under conditions that stimulated expression of *vir* genes and the *repABC* operon, cells bearing pTi15955 were at a marked competitive disadvantage (Platt et al., 2012a). This demonstrates that the expression of these genes is highly costly indicating that the infection of plant hosts comes at a significant cost to the infecting agrobacteria.

**FIGURE 1:**
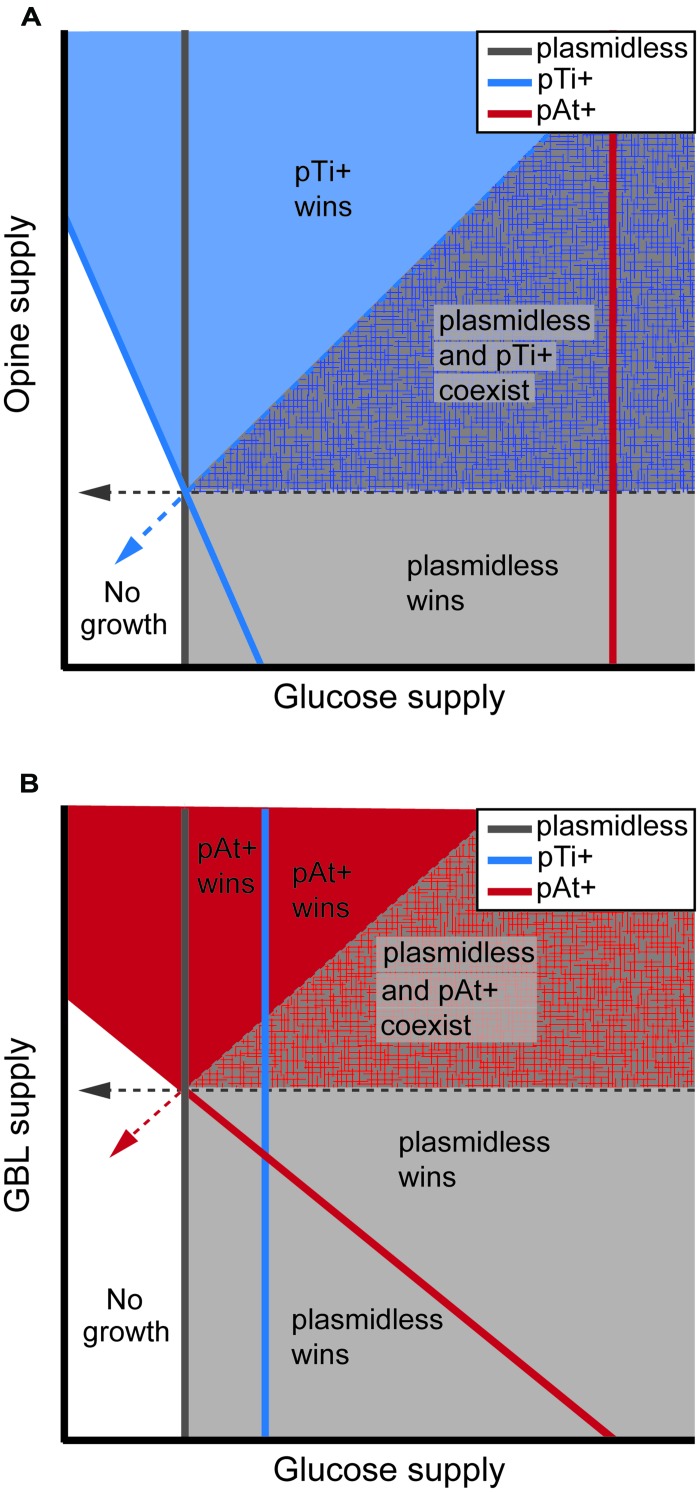
**Model predictions for the outcome of resource competition between plasmidless, Ti plasmid bearing, and At plasmid bearing agrobacteria for opines and glucose (A) and for γ-butyrolactone (GBL) and glucose (B).** Solid lines represent the minimum resource levels required for growth, while dashed arrows represent the consumption vectors of the indicated strain. Plasmidless agrobacteria can catabolize glucose while pTi+ cells can catabolize both glucose and opines and pAt+ cells can catabolize both GBL and glucose. Because At plasmids are likely much more costly than Ti plasmids, the minimum amount of glucose needed to support a population of pAt+ cells is considerably greater than that of Ti+ cells (compare the x-intercepts of the zero-net-growth-isoclines of these strains). Zones of each phase plane are shaded according to which genotype or genotypes are able to persist in the corresponding environments despite resource competition with the other strains. Note that the lower left zone of each phase plane represents environments in which resource levels are too low to support growth of any of the genotypes. See Morton et al. (2014) for a more thorough model analysis that includes competition by genotypes bearing both a Ti plasmid and an At plasmid and Platt et al. (2012b) for an analysis of competition of genotypes bearing a Ti plasmid and avirulent, freeloading strains whose plasmids only contain the opine catabolism genes.

Key benefits associated with the Ti and At plasmids stem from their conferring the ability to catabolize nutrients. Consequently, resource-consumer competition models provide a useful way to describe competition among genotypes that vary in the plasmids they harbor. **Box 1** provides an overview of how the predictions of these models can be graphically represented. As articulated in the “opine concept,” the primary benefit of plant pathogenesis for pathogenic agrobacteria comes in the form of the opines produced by the infected plant (Guyon et al., 1993; Oger et al., 1997; Savka and Farrand, 1997; Mansouri et al., 2002). Opines exuded by infected plants provide a nutrient source that can promote the growth of opine catabolic bacteria in the rhizosphere. For example, octopine availability can shift the outcome of resource competition between pathogenic agrobacteria harboring pTi15955 and avirulent strains lacking the plasmid (**Figure 1A**; Platt et al., 2012b). In this way, opines arise from the costly action of agrobacteria infecting host plants. Upon exudation by the plant, opines are available for any opine catabolic bacteria, which can include agrobacteria harboring opine catabolic plasmids as well as other rhizosphere bacteria (Moore et al., 1997). In addition to the benefits associated with opine catabolism following infection of a plant host, Ti plasmids also confer the ability to detoxify phenolics or even the use of these phenolics as nutrient sources (Brencic et al., 2004).

**Box 1. The predictions of resource consumer competition models can be graphically represented and interpreted using two-dimensional plots in which the graphical axes represent concentrations of the two resources for which the competitors compete.**

Each species’ population grows in environments where the supply of resources is sufficiently high to support its growth. In the heuristic example shown, the solid line corresponding to each species represents environments where that species’ population neither grows nor declines in size. Consequently these lines are referred to as zero-net-growth isoclines, or ZNGIs. All environments above the line have sufficient resource levels to support population growth of the species, while all environments below the line cannot support population growth of that species. In this example, the two resources are substitutable, such that each species can maintain an equilibrium population in environments with exclusively one or the other resource (e.g., the ZNGI x- and y- intercepts) or combinations of the two resources (e.g., the points on the line between the intercepts). The shape and position of ZNGIs can vary depending on the attributes of the consumer and the way in which resources influence the growth of that species. The dashed arrows are vectors representing the rate at which the associated species consumes each of the resources.

Predicting the establishment of these species in some environments is straightforward. All points in zone A are under the ZNGIs of both species 1 and species 2. Since populations of neither species can grow in zone A, then populations of neither will persist in environments with those combinations of resource levels. Similarly since populations of only species 1 can grow in zone B and only species 2 can grow in zone F, only those species can establish populations in those environments. In these models, one competitor displaces the other if growth of its population drives resource levels below the minimal needs of the other species. This occurs in zones C and E with species 1 driving resource levels below the minimal needs of species 2 in zone C, and species 2 driving resource levels below the minimal needs of species 1 in zone E. In this example, both species stably coexist in zone D since neither species drives resource levels below the minimal needs of the other species. See Tilman (1980, 1982) for a more detailed analysis of a variety of competitive scenarios.

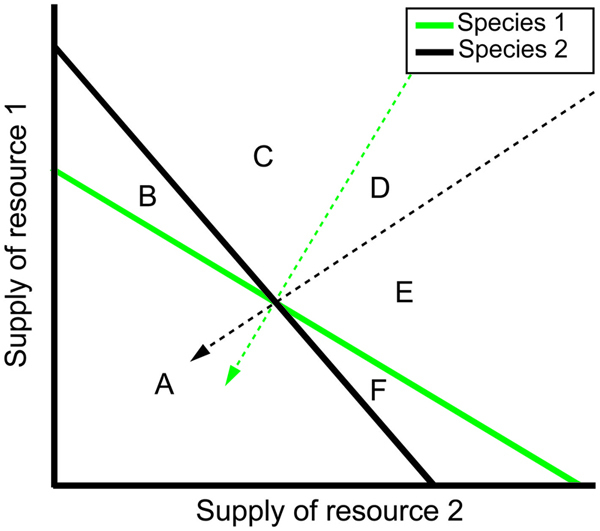

In contrast to the Ti plasmid whose carriage cost is minimized under conditions where plasmid benefits are limited, the At plasmid carriage cost as measured in the C58 nopaline type strain, is high (Morton et al., 2014). While the reason for the observed high cost has yet to be determined, there are several potential explanations. For example, this At plasmid is self-conjugal, and unlike the majority of previously characterized conjugal megaplasmids, conjugation of pAtC58 is constitutive (Chen et al., 2002). That is, pAtC58 conjugates at the same frequency under a range of laboratory conditions (although it is possible that there is as yet unrecognized environmental control of this conjugation). This data correlates directly with expression analyses of the genes encoding the pAtC58 conjugal pilus, the *avhB* operon (Perez-Mendoza et al., 2005; Morton et al., in preparation). This operon encodes a Type IV secretion system responsible for pAtC58 conjugation and homologous to the VirB pilus of Ti plasmid, which mediates transfer of the T-DNA during plant infection (Chen et al., 2002). Expression of this Ti plasmid encoded system has been demonstrated to be energetically quite expensive (Platt et al., 2012a). However, given that a truncated form of the plasmid lacking the *avhB* genes still confers a significant cost to its host cells, conjugation is likely not the sole contributor to the high cost of the plasmid (Morton et al., 2013). In addition to the AvhB system, there are multiple ATP binding-cassette (ABC) transporters encoded on the plasmid. As transmembrane proteins requiring energy in the form of adenosine triphosphate (ATP) to transfer molecules across membranes, these transporters could explain a portion of At plasmid costs. Despite its high cost, pAtC58 is extremely difficult to cure and attempts to do so frequently result in genomic restructuring (Morton et al., 2014). It is perhaps the high cost of the plasmid, coupled with mechanisms that ensure its maintenance (i.e., toxin–antitoxin systems) that drives selection for the observed deletions (Morton et al., 2013).

In addition to plasmid-encoded stability functions, pAtC58 prevalence in the environment is in part explained by the catabolic benefits it provides to its host bacteria (Morton et al., 2014). The competitive advantage in the rhizosphere garnered by strains of *A. tumefaciens* harboring pAtC58 is likely attributed to the plasmid-conferred ability to catabolize such molecules as GBL and DFG as a sole carbon source (**Figure 1B**; Baek et al., 2000; Morton et al., 2014). DFG, also known as santhopine, is an Amadori compound found in decaying plant material in addition to the tumors of plants transformed by chrysopine-type strains of *A. tumefaciens*. DFG catabolism is encoded by a set of genes located adjacent to the *repABC* operon on pAtC58 called *socR* and *socABCD* (Baek et al., 2003). In addition to its prevalence in the rhizosphere, DFG is formed during catabolism of agropine (AGR) and MOP, both functions encoded by octopine/mannityl opine-type Ti and Ri plasmids (Hong and Farrand, 1994). Oxidation of MOP (taken up directly from the environment or formed by the de-lactonization of AGR) results in the formation of DFG (Kim and Farrand, 1996). The uptake and catabolism of MOP and AGR is conferred by the products of the adjacent genes, *mot, ags*, and *moc* (Hong et al., 1997; Oger et al., 1998).

Strains harboring pAtC58 are able to grow using GBLs or related compounds as a sole carbon source. This growth is dependent on a functional *blcABC* operon—previously named *attKLM*, as mentioned above (Khan and Farrand, 2009). GBLs are common plant exudates and thus pAtC58 confers the ability to catabolize resources associated with the rhizosphere environment. Many soil bacteria rely on quorum-sensing (QS) to monitor population density and regulate community behaviors accordingly (Fuqua et al., 2001). Several soil bacteria including streptomycetes produce and employ GBL derivatives as quorum sensing signaling molecules (Du et al., 2011). The *blcC* gene, encoding a lactonase, and its homologs have received considerable attention due to their potential quorum quenching effects in degrading acyl homoserine lactone (AHL) signal molecules. The BlcC lactonase can effectively degrade AHLs to form *N*-acyl-homoserines, rendering them inactive as quorum sensing signals. The transcriptional repressor *blcR* is divergently transcribed from the *blcABC* operon. Null mutants in *blcR* fail to accumulate the Ti plasmid encoded AHL, 3-oxo-C8-HSL, due to overexpression of *blcC* (Zhang et al., 2002).

Break-down products of GBLs are intermediates of the tricarboxylic acid (TCA) cycle, which can also induce *blcABC* expression by causing the dissociation of BlcR from the *blcABC* promoter, thereby inhibiting the accumulation of AHL under more natural conditions. The biological relevance of this, however, is still unclear and the subject of considerable debate. There is convincing evidence demonstrating that artificial induction of this operon during infection will cause an initial delay in tumorogenesis, but that over time these effects are negligible (Khan and Farrand, 2009). Additionally, although the *blcABC* operon confers GBL catabolism, GBL is only a minor inducer of expression of these genes. Strong expression requires the presence of the GBL breakdown products SSA, GHB, or GABA, a non-protein amino acid expressed in plant tissues in association with stress or mechanical damage (e.g., wounding; Khan et al., 2007). Please refer to Lang and Faure (2014) for a more extensive discussion on this topic.

## ECOLOGICAL CONTEXT OF Ti and At PLASMIDS

The rhizosphere is the soil at the interface of plant root tissue. Here, plant roots influence the conditions of the soil to create a dynamic environment that is rich in microbial life (Badri et al., 2009). Because of this diversity, the rhizosphere is the seat of intense resource and interference competition among the resident microbes (Raaijmakers et al., 2009). Further, the interactions that occur between plants and the rhizosphere microorganisms as well as microbe-microbe interactions have large effects on the dynamics of both plant and microbial communities (Bever et al., 2012; Philippot et al., 2013). Agrobacteria must contend with intense competition with other agrobacteria and rhizosphere microbes associated both with healthy and with crown gall-diseased plants.

The plant tumor environment is remarkably diverse and can include several different types of opine catabolic microorganisms. Though opines are relatively uncommon metabolites that provide nutrients promoting the growth of the pathogenic agrobacteria, several other soil bacteria have the ability to catabolize specific opine species (Tremblay et al., 1987; Beauchamp et al., 1990; Bergeron et al., 1990; Nautiyal and Dion, 1990; Nautiyal et al., 1991; Moore et al., 1997). Additionally, colonization by opine catabolic, avirulent agrobacteria and the *de novo* evolution of avirulent freeloaders via loss of virulence functions have both been observed (Llop et al., 2009). Pathogenic agrobacteria are likely to be at a competitive disadvantage to these avirulent, opine catabolic agrobacteria that do not pay the costs associated with harboring the intact Ti plasmid or cooperatively infecting plants, raising the possibility that the pathogen may become displaced from the tumor environment it elicited (Platt et al., 2012a,b). While the competitive disadvantage of virulent strains against avirulent freeloaders may be sufficient to explain their exclusion from diseased environments, pathogenic agrobacteria also must contend with even more overt competitive mechanisms such as bacteriocin-mediated interference competition with other rhizosphere bacteria (e.g., Kim et al., 2006). In this way, K84 and similar opine catabolic, avirulent strains are able to highjack the crown gall environment engineered by pathogenic agrobacteria, hence K84 has utility as a biocontrol agent for certain types of crown gall disease (Farrand et al., 2007).

The non-protein amino acid GABA is involved in a wide variety of cellular responses that extends across kingdoms. In animals, GABA acts as a neurotransmitter and in plants and bacteria, the molecule is usually involved in biotic and abiotic stress responses. GABA is produced in wounded plant tissues as part of a complex defense response, where this molecule is taken up in *A. tumefaciens*. Uptake has been shown to require an ABC transporter BraDEFG (Atu2424- Atu2427) and a periplasmic binding protein (Atu2422; Chevrot et al., 2006; Haudecoeur et al., 2009b). GABA resembles break-down products of the QS signal 3-oxo-C8-HSL which is responsible for activating Ti plasmid replication and conjugation through its interaction with the regulator, TraR. Regulation of GABA uptake in A. *tumefaciens* has recently been shown to primarily occur via the sRNA AbcR1 (Wilms et al., 2011). This sRNA was found to destabilize the transcript of the proline/GABA periplasmic binding protein, Atu2422. In stationary cultures, strains deficient in AbcR1 accumulate Atu2422 and GABA to a much higher level than WT cells. It was proposed that the sRNAs serve to reduce levels of the Atu2422 transcript and minimize intracellular levels of GABA. Exclusion of GABA from the cell would prevent BlcC-mediated quorum quenching and maintain physiological AHL-regulated Ti conjugation and expression of virulence genes (Wilms et al., 2011, 2012). Additional regulation of GABA uptake has been suggested through proline as it competes with GABA for binding to the periplasmic protein, Atu2422, which is required for uptake of both molecules (Haudecoeur et al., 2009a). It is unclear, however, what role proline accumulation plays in the plant–*Agrobacterium* interaction.

While GABA stimulates expression of *blcABC* and degradation of QS signals, it has also been shown that agrocinopines stimulate production of another, Ti-encoded, lactonase, AiiB homologous to BlcC, which may be involved in the reduction in accumulation of 3-oxo-C8 HSL and a modulation of QS-mediated Ti plasmid conjugation (Liu et al., 2007). AiiB was shown to be excluded from AccR-mediated regulation, meaning that it acts independent of 3-oxo-C8 HSL levels (Haudecoeur et al., 2009b). AiiB and BlcC are thus regulated by separate pathways, suggesting they play distinct roles in the degradation of QS signals during *A. tumefaciens* plant interactions and pathogenesis.

For nopaline-type *A. tumefaciens* strains, the agrocinopines produced by the infected plant control expression of many genes, primarily through the regulator, AccR. These genes include the *arc* genes (including the *traR* gene encoding the AHL-responsive quorum sensing regulator), the *acc* genes and, more recently discovered, the *noc* genes through *nocR*. TraR is directly responsible for the expression of T4SS genes for the conjugal transfer of the Ti plasmid, meaning that agrocinopines, through AccR, control dissemination of this replicon. Similarly, it was recently shown that AccR also regulates expression of pAtC58’s *rctB*, orthologs of which have been shown to be involved in control of symbiotic plasmid conjugation in related rhizobia *R. etli* and *S. meliloti* (Perez-Mendoza et al., 2005; Lang et al., 2013; Nogales et al., 2013). A recent paper demonstrated that AccR regulates the conjugation of both the Ti and At plasmids by repressing transfer of both replicons in the absence of agrocinopines (Lang et al., 2013). These results suggest that conjugation of co-resident Ti and At plasmids may be enhanced in the tumor environment. This would potentially result in co-transfer or competitive transfer of the At and Ti plasmids.

## ECOLOGICAL AND EVOLUTIONARY CONSEQUENCES OF INTERACTIONS BETWEEN THE Ti AND At PLASMIDS

Pathogenic agrobacteria pay a high cost to translocate the T-DNA into the plant’s genome. However the resulting infection can benefit other individuals such as neighboring opine catabolic agrobacteria making this a cooperative behavior (Platt and Bever, 2009; Gardner and West, 2010; Platt et al., 2012a). The primary benefit of agrobacterial pathogenesis stems from the catabolism of public good nutrients, the opines that infected plants produce (Guyon et al., 1993; Oger et al., 1997; Savka and Farrand, 1997; Mansouri et al., 2002; Platt et al., 2012b). The competitive advantage of cheating genotypes threatens the evolutionary stability of any cooperative behavior (Hamilton, 1964a,b). Due to the high cost associated with infecting plants there is a strong selective pressure favoring avirulent, freeloading genotypes that retain the ability to access these benefits by catabolizing opines (Platt et al., 2012b). Non-pathogenic, opine-catabolic agrobacteria have frequently been isolated from plant crown gall tumors (Merlo and Nester, 1977; Nautiyal and Dion, 1990; Bouzar et al., 1993; Belanger et al., 1995). Growth of laboratory cultures of several strains of *A. tumefaciens* in the presence of *vir*-inducing phenolic compounds results in the origin and rapid spread of mutated strains that have generated plasmids incapable of conferring virulence (Fortin et al., 1992, 1993; Belanger et al., 1995). The evolution of avirulent agrobacteria from a pathogenic strain has also been observed in plant tumor tissues; however in this study non-pathogenic agrobacteria more often colonized the plant from the environment (Llop et al., 2009). This result is perhaps surprising given the apparent rapid rate of evolution of avirulent plasmids in the lab. Llop et al.’s (2009) observations suggest that avirulent freeloading is a successful and persistent strategy in nature and motivates future work examining the relative importance of mutation and colonization to the success of avirulent freeloaders.

Regardless of the origin, the ability of avirulent, opine-catabolic agrobacteria to invade plant tumors elicited by pathogenic agrobacteria, poses a significant challenge to the persistence of the pathogen. The costs of the Ti plasmid put the pathogen at a competitive disadvantage when opines are not present (**Figure 1**). Further, competition with avirulent, opine-catabolic agrobacteria threatens the persistence of the pathogen in the host environment as well (Platt et al., 2012b). Thus, non-tumor soils are predicted to be a sink population for the pathogen due to competition with saprophytic agrobacteria, while freeloading avirulent bacteria can competitively displace pathogens from tumor soils. The persistence of agrobacterial pathogens, then, critically depends on the pathogen’s neighbors tending to be other pathogens such that the individuals that pay the cost of infecting the plant have at least a transient exclusive access to the plant tumor environment. The conditions for pathogen persistence are relaxed if the tumor environment can support a larger population size than the healthy plant root (Platt et al., 2012b). The resolution of this tension will determine the prevalence of pathogenic strains in agrobacterial populations and therefore the disease incidence on the plant host.

Although the At and Ti plasmids are central drivers of the ecological dynamics of *A. tumefaciens* strains, there is a clear lack of knowledge regarding their frequency and distribution in nature, particularly in non-tumor environments. The spatial and temporal heterogeneity of the soil makes it difficult to define any particular microhabitat, for which the selective pressures affecting each plasmid’s fitness are expected to vary (Platt et al., 2012a,b; Morton et al., 2014). Natural populations of pathogenic agrobacteria fluctuate seasonally, change across years, and can persist several years in soils that lack readily observable plants exhibiting crown gall disease (Bouzar et al., 1993; Krimi et al., 2002). In some cases the frequency of Ti plasmid bearing cells in nature has been observed to decline over time in the absence of opine-producing tumors of infected plants (Krimi et al., 2002). In contrast to this, the same study observed several instances where cells bearing a Ti plasmid appeared to have a competitive advantage over cells lacking a Ti plasmid, despite the absence of a crown gall tumor. Such observations may result from benefits conferred by chromosomal genes, the presence of cryptic tumors, or yet uncharacterized benefits conferred by Ti plasmids (Krimi et al., 2002). This highlights the importance of further work establishing the variety of factors driving the dynamics of natural agrobacterial populations.

Genomic characterization of natural isolates reveal that At plasmids are very commonly found in association with a Ti plasmid and strains that lack a Ti plasmid, frequently still carry an At plasmid. This is perhaps explained by the array of rhizosphere-specific catabolic functions encoded by At plasmids (DFG, GABA, and GBL). One greenhouse study shows that a strain with an At plasmid outcompetes an isogenic strain lacking the plasmid in the rhizospheres of infected plants (Morton et al., 2014). When rhizosphere-specific resources are depleted, the direction of the competitive interaction is reversed.

In addition to the direct effects of resource competition, the ecological dynamics of *A. tumefaciens* genotypes are determined by intracellular interactions. One example of this is the elevated expression of Ti plasmid virulence genes in cells that harbor a laboratory-evolved At plasmid (Morton et al., 2013). Competitions between pairs of isogenic C58 plasmid genotypes (plasmidless, carrying At plasmid, carrying Ti plasmid, and carrying both At and Ti plasmids) revealed genotype-specific interactions that differ significantly from what would be predicted based on independent plasmid costs. For example, the cost of the two plasmids together is non-additive (Morton et al., 2014). Incorporating these empirically determined plasmid-specific costs and benefits into a resource consumer model of competition shifted the predicted outcomes at equilibrium such that cells harboring an At plasmid are expected to dominate environments with a broader range of resource supply conditions (Morton et al., 2014). As a facultative pathogen *A. tumefaciens* cells occupy a range of resource environments. The environment-specific costs and benefits of the At and Ti plasmids coupled with the observed non-additive costs suggest that in a temporally dynamic and spatially structured environment, strains with both plasmids could have a competitive advantage relative to plasmidless or single plasmid genotypes.

## SUMMARY AND FUTURE DIRECTIONS

Agrobacterial plasmids play a central role in the ecology and evolution of the bacteria that harbor them. These plasmids confer a wide range of phenotypes including the ability to infect plant hosts, catabolize plant-produced nutrients, and produce bacteriocins mediating interference competition with other rhizosphere bacteria. In this review we have described the wide range of ecological and molecular interactions that shape the evolution and ecological dynamics of agrobacterial plasmids.

Environmental resource levels play a key role in determining the relative costs and benefits associated with many agrobacterial megaplasmids (e.g., **Figure 1**). Consequent impacts on the competitive ability of plasmid-harboring strains thereby influence the spread and decline of these plasmids. A significant challenge remains in integrating these local scale competitive interactions into a meta-community framework that would predict population virulence levels and disease incidence. This effort will require additional information on other aspects of agrobacterial life-history, including their dispersal and dormancy. A secondary level of questions relates to the environmental benefits of the different variants of virulence plasmids. Do, for example, agrocinopine Ti plasmids confer a competitive advantage in P-limited environments while Ti plasmids conferring catabolism of secondary amine derivatives confer a competitive advantage in N-limited environments?

Conjugal transfer is a second factor determining the evolution of agrobacterial megaplasmids as it allows colonization of novel genetic backgrounds. An open question for future research is to what degree conjugation also impacts competition among variant agrobacterial plasmids. The fitness of conjugal plasmids is composed of contributions from both its vertical transmission during reproduction of the host cell and its horizontal transmission during conjugation. Because of this impact on plasmid fitness, conjugation may have a role in shaping the competitive ability of these plasmids.

Conjugation may also play a role in allowing for interactions between competing plasmids mediated by the toxin-antidote systems that they encode (Cooper and Heinemann, 2000, 2005). Several agrobacterial plasmids harbor such systems (Yamamoto et al., 2007, 2009), however their role in mediating interactions among competitor plasmids is yet uncharacterized and will be an interesting question for future research. Entry exclusion systems may play a role in mediating these affects as they provide a way to prevent entry of the host bacterium by competitor plasmids that may evict the resident plasmid via a toxin-antidote system. An intriguing aspect of the Ti plasmid system is the hierarchical regulation of conjugation by opines and a quorum sensing system (Lang and Faure, 2014, this issue). This mechanism of gene regulation is responsive to both the levels of available resources that can be catabolized by Ti plasmid bearing cells, and the density of those cells. Why conjugation is regulated in this way, as it relates to plasmid fitness and competitive ability, is an exciting question for future work.

One of the strengths of agrobacteria as a model system for such questions is the ability to readily integrate lab, greenhouse, and field studies of this microorganism. Our understanding and ability to manipulate relevant environmental and genetic factors allows for experimental dissection of forces influencing the ecological success and evolution of these plasmids in the laboratory and more realistic greenhouse settings. Such work can be coupled with examination of the plasmid dynamics in field populations to provide a clear picture of what drives the success of agrobacterial megaplasmids in nature. These types of studies also address more general issues such as how the joint effects of dynamics in host and non-host environments influences the ecology and evolution of diseases caused by facultative pathogens. While many important diseases are caused by facultative pathogens, the dynamics of these pathogens occurring in non-host environments is poorly incorporated into most models of disease epidemiology and evolution. Consequently the experimental tractability and the established environmental context dependence to the fitness of agrobacterial pathogens makes these bacteria powerful model systems for studying the intersection of microbial ecology and disease dynamics for facultative pathogens.

## Conflict of Interest Statement

The authors declare that the research was conducted in the absence of any commercial or financial relationships that could be construed as a potential conflict of interest.
